# Optimal Pricing Decisions for a Low-Carbon Supply Chain Considering Fairness Concern under Carbon Quota Policy

**DOI:** 10.3390/ijerph18020556

**Published:** 2021-01-11

**Authors:** Hao Zou, Jin Qin, Bo Dai

**Affiliations:** 1 School of Traffic and Transportation Engineering, Central South University, Changsha 410075, China; zouhaocm@csu.edu.cn (H.Z.); qinjin@csu.edu.cn (J.Q.); 2 School of Business Administration, Hunan University of Finance and Economics, Changsha 410205, China; 3 School of Management, Hunan University of Technology and Business, Changsha 410205, China; 4 Laboratoire d’optimisation des Systeme Industriels, Institut Charles Delaunay, Universite de Technologie de Troyes, 10010 Troyes, France

**Keywords:** low-carbon supply chain, fairness concern, carbon quota policy, decision analysis

## Abstract

This research investigates the effect of fairness concerns on a sustainable low-carbon supply chain (LCSC) with a carbon quota policy, in which a manufacturer is in charge of manufacturing low-carbon products and sells them to a retailer. The demand is affected by price and the carbon emission reduction rate. The optimal decisions of pricing and carbon emission reduction rate are analyzed under four decision models: (i) centralized decision, (ii) decentralized decision without fairness concern, (iii) decentralized decision with manufacturer’s fairness concern, (iv) decentralized decision with retailer’s fairness concern. The results indicate that the profits in the centralized LCSC are higher than those in the decentralized LCSC with fairness concern. If a manufacturer pays close attention to fairness, the fairness concern coefficient will reduce the carbon emission reduction rate and the profit of the LCSC and increase the wholesale price and the retail price of the product. If a retailer pays close attention to fairness, and the preference of consumers for a low-carbon product is low, the fairness concern coefficient of the retailer increases the total profit of the LCSC and decreases the carbon emission reduction rate and retail price of the product. Otherwise, if the preference of consumers for a low-carbon product is great, the fairness concern coefficient of the retailer would lead to a lower retail price compared with the retail price in the centralized decision and decrease the total profit of the LCSC.

## 1. Introduction

The environmental problems caused by the greenhouse effect have become more and more serious [1]. Global warming brings a huge pressure to human survival and health and has aroused widespread concern around the world [2]. To this end, many governments have implemented environmental policies such as carbon taxes and cap and trade [3]. In practice, Canada and Australia introduced carbon taxes in 2008 and 2012 respectively [4]. Compared with carbon tax, cap and trade is also conducive to saving energy and reducing emissions. For instance, both the European Union’s emissions trading scheme [5] and the China’s cap-and-trade scheme [6] are successful solutions. In a cap-and-trade scheme, companies receive a carbon quota from the government and trade the quota by taking into account their emissions. Since 2012, China has piloted carbon trading in seven cities, which includes Beijing, Shanghai and Shenzhen, etc. Meanwhile, the Chinese government formally implemented a carbon trading policy in 2017 [7]. Therefore, carbon emission reduction has become an important goal to achieve high-quality economic development in China.

In reality, governments guide consumers to buy green products by using carbon labels. For example, China’s energy labeling program was significant in guiding consumers to buy low-carbon and environmentally friendly products, which was launched in 2005 and involved household appliances, automobiles and other industries [8]. Through the government subsidies, China’s energy labelling program has not only encouraged companies to produce low-carbon products, but also changed consumers’ purchase preferences. Adaman et al. [9] showed that consumers were willing to pay more for environment-friendly products. Meanwhile, with the implementation of more and more environmental regulations and the development of low-carbon technologies, many manufacturing enterprises are committed to the production of green products to improve their competitiveness, which help directly reduce carbon emissions [10,11]. For instance, a California clothing company has produced green products for many years [12], and Gree Electric of China has focused on energy efficient product design and innovation and invested more than $1 billion in carbon reduction technologies in 2008 [8].

In order to meet the growing low-carbon demand of consumers, the upstream enterprises of the supply chain have to accelerate the development of emission reduction technologies, and the downstream enterprises have to increase the promotion of low-carbon products. This will increase the operating costs of the supply chain. Thus, low-carbon supply chain (LCSC) management has become an important research topic in recent years.

Empirical research shows that the members in a supply chain have fairness preferences, which are often referred to fairness concerns. Specifically, they focus not only on maximizing their own profits, but also on the fair results of profit distribution in the supply chain [13]. For example, Xuzhou Wanji Trading Co., Ltd. from Xuzhou of China terminated its distribution business with P&G from Cincinnati of America in 2010 because it believed that P&G’s products were unreasonably priced. In 2010, Haier Electric was committed to the development and production of low-carbon products, while retailers such as Gome and Suning carried out product sales. However, as the manufacturer was in a strong position, Haier was more concerned with its own interests, which caused the unfair feelings for Gome and Suning [14]. In the face of unfair treatment of peers, the fairness of enterprises plays an important role in their decision-making [15]. Therefore, fairness is an important factor that should be considered when manufacturing enterprises invest in emission reduction and supply chain operation management [4]. Studies have shown that fairness concern not only affect the price strategies and efficiency of supply chains [15,16], but also can encourage manufacturers to make more use of green technologies [17]. Manufacturing enterprises adopt low-carbon technology to reduce carbon emissions, which will increase costs. As rational decision-makers, manufacturers may deliberately raise wholesale prices in order to share emission reduction costs, which will lead to the unfair treatment of downstream retailers and ultimately reduce their incentives [14]. Therefore, the decision-makers in the supply chain not only consider their own profits, but also consider the fairness among members. Therefore, it is very important to study the pricing decisions in LCSCs with the members’ fairness concerns.

To our best of knowledge, only a few papers have simultaneously studied the impact of carbon emission limit and fairness concern on the optimal prices and carbon emission strategies of members in low-carbon supply chains [15,18,19]. However, most studies rarely incorporate both carbon quota policy and fairness concern into the decision-making process of LCSCs. Different from these studies, our research attempts to solve the following questions: (1) What is the difference between fairness neutrality and fairness concern in pricing decisions? (2) Under the carbon quota policy, how do the fairness concerns of different members in the supply chain affect the pricing of low-carbon products and the profits of all members? (3) How does the consumers’ low-carbon preference for products affect the pricing of low-carbon products and the profits of all members in the supply chain? Therefore, this paper considers a LCSC with a cap-and-trade system and establishes a master-slave decision-making model by using game theory, where a pricing decision-making problem under the fairness concerns of both manufacturers and retailers is studied. The purpose of this study is to provide a theoretical basis and decision support for the low-carbon supply chain pricing processes considering the fairness concerns of its members. The novelty of this paper can be concluded as follows. Firstly, the carbon quota policy and the fairness concern of members are considered in the pricing decision-making process of the low-carbon supply chain. Secondly, the consumers’ preferences for low-carbon products are discussed in the above decision-making process. Thirdly, by using dynamic game theory, four pricing decision-making models for low-carbon supply chains (LCSCs) are proposed and analyzed. The rest of the paper is organized as follows. Section 2 introduces a comprehensive review of carbon quota and fairness concern based on LCSCs. Section 3 describes the notation and assumptions of the model. Section 4 analyzes different models. Section 5 presents numerical analysis and sensitivity analysis. Section 6 introduces the conclusion and research prospects.

## 2. Literature Review

The previous studies related to our study can be classified to three categories, which will be reviewed in the same way like the references [20,21,22,23]. The first category is the impact of carbon policy on low-carbon supply chain management decisions. The second category is the impact of fairness on supply chain management decisions. The third category is the methods adopted in supply chain management decisions.

### 2.1. The Impact of Carbon Policy on Low-Carbon Supply Chain Management Decisions

Compared with carbon tax control, total emission control has become a hot topic in academic research. The operational management research based on cap-and-trade systems includes pricing, inventory and emission reduction decisions [7,24]. Hua et al. [25] discussed the carbon footprint based on the mechanism of carbon emission, which analyzed the impact of a carbon emission cap and carbon price on supply chain decision-making. Du et al. [26] studied the impact of carbon emission and trading policies on decision-making of a supply chain under a single period of random demand. Xu et al. [27] discussed the joint production of multi-commodity manufacturing enterprises which considered the total carbon emission control and carbon tax control. Wang et al. [28] established a retailer-dominated carbon emission reduction game model and studied the carbon emission reduction under the dominant position of retailers and the balance of power. Wang et al. [29] constructed a mathematical model and found that production planning was positively correlated with carbon quota and the processing cost was negatively correlated with carbon emission rate. Halat and Hafezalkotob [30] constructed a mathematical model that considered the inventory costs and carbon emissions of a supply chain and explored the effects of coordination mechanisms and carbon regulations on inventory costs, carbon emissions and government objectives. Liu [31] studied the pricing and coordination mechanism of supply chains based on big data and the impact of target advertising on demand.

### 2.2. The Impact of Fairness on Supply Chain Management Decisions

The above research on operations and management of LCSCs assumed that decision-makers were completely self-interested. However, in real life, if there is a profit allocation among participants, they are concerned whether they are treated fairly. In the earlier literature, Cui et al. [32] and Ho et al. [33] considered the fairness concern in supply chain contracts. Loch et al. [34] found that supply chains can still be coordinated when unfair aversion occurs to retailers. Current studies about fairness concern mainly deal with supply chain pricing and system coordination. Shi et al. [35] discussed the supply chain pricing problem that considered the fairness concern of channel members. However, few studies have integrated fairness into LCSCs. Zhou et al. [14] studied the impact of retailers’ fair attention on decision-making and the coordination of supply chain optimization. However, their study did not take into account the impact of carbon quotas. Liu et al. [4] found that carbon tax regulation promoted manufacturers to improve product sustainability by examining the impact of manufacturers’ and retailers’ fairness concern on their production. In contrast, we focus on the impact of cap-and-trade regimes. Based on the retailer’s fairness concern, Li et al. [15] found that the retailer’s fairness concern had a significant negative influence on manufacturer’s price and decision-making. Zhang et al. [18] discussed the optimal decision-making scheme of the supply chain in four decision-making backgrounds, but they only considered retailer’s fairness concern. Li et al. [19] studied the dynamic price game of the low-carbon closed-loop supply chain system through considering the members’ fairness concern behavior. Li et al. [36] studied the low-carbon double-channel supply chain and discussed the impact of fairness concern behavior on the price stability. Han et al. [37] built game models to show that consumers’ preference for low-carbon products is good for the operation of the supply chain.

### 2.3. Research Methods Adopted in Supply Chain Management Decisions

Zhao et al. [38] used game theory to study the pricing and decision-making strategies of supply chain companies under the dual criteria of reducing carbon emissions and maximizing profits under the two situations of government supervision and voluntary emission reduction of supply chain enterprises. Du et al. [39] used the Nash bargaining solution as a fair reference value to study the impact of fair behavior on supply chain pricing and decision-making. Du et al. [26] analyzed the low-carbon supply chain under carbon trading through game theory and explored the impact of low-carbon awareness consumers on supply chain operations. They found that low-carbon awareness of consumers can effectively promote carbon reduce production and increase the profitability of the supply chain. Zu et al. [40] used the Stackelberg differential game model to analyze the low-carbon emission reduction decision-making process of the two-level supply chain under three progressive environmental management levels (government low-carbon intervention, enterprise supply chain coordination, and consumer environmental protection and low-carbon awareness).

In summary, some studies have considered the fairness concerns of the members in the pricing decision-making of a low-carbon supply chain. However, they rarely incorporate carbon quota policies and fairness into the low-carbon supply chain decision-making process at the same time. The study of Liu et al. [4] was most related to our paper. They found that carbon tax control can encourage manufacturers to improve the sustainability level of products. In contrast, our research focuses on the impact of total volume restricted carbon trading systems and considers consumers’ preferences for pricing decision-making of low-carbon products, which is different from all previous studies. To better observe the behaviors of manufacturers and retailers in the pricing decision-making of the supply chain, dynamic game theory is used in this study.

## 3. Problem Description

We design a two-echelon low-carbon supply chain (LCSC) which includes a retailer and a manufacturer. The manufacturing process produces carbon emissions. At the beginning of each year, the government gives a free carbon quota to the manufacturer. If the manufacturer’s carbon emissions exceed the quota, it will have to buy carbon credits from a carbon trading market, and when the manufacturer’s carbon emissions are lower than the government’s quota, it can sell the excess carbon credits.

**Figure 1:**
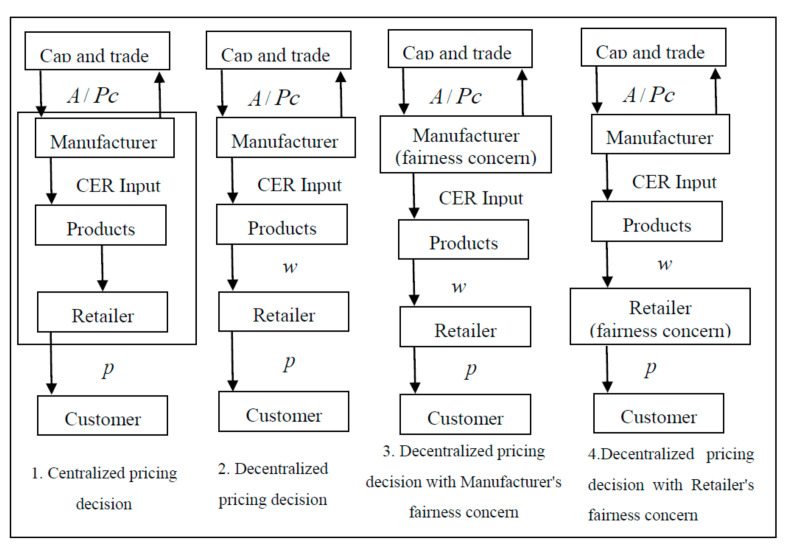
The four cases of pricing decisions.

Firstly, the centralized pricing decision-making and decentralized pricing decision-making processes of the LCSC are studied. Then, the decentralized pricing decision-making process of the LCSC is studied respectively, where dynamic game theory is applied. A flow chart of the decision-making processes in the LCSC is shown in Figure 1. The government first gives the manufacturer a certain carbon quota (A). If the amount of carbon produced by the manufacturer exceeds *A*, the manufacturer will buy the carbon emission right in the trading market with a price $p_{c}$, otherwise manufacturers would sell carbon credits with a price $p_{c}$. To investigate the optimal pricing decisions by considering fairness concern under carbon quota policy, four cases of pricing decisions will be discussed, which are shown in Figure 1. In case 1, a centralized pricing decision for the LCSC is made in which the manufacturer and retailer act as a whole to face the sales market and sell the product to the customer with a price p. In case 2, a decentralized pricing decision for the LCSC is made where the manufacturer first sell the product to the retailer with a wholesale price w and then the retailer sells the product to the customer with a price p. In case 3 and 4, a decentralized pricing decision is made for the LCSC by considering the fairness concern of the manufacturer and the fairness concern of the retailer, respectively.

## 4. Models and Analysis

In the studied low-carbon supply chain (LCSC), the manufacturer produces green products and invests in carbon reduction, and the retailer sells green products and has a low-carbon preference. The relationship between the two submits to the master–slave game in which the manufacturer takes the lead.

### 4.1. Notations and Assumptions

For lucidity and simplicity, the parameters and core variables are expressed in Table 1.

This paper assumes the following:

(1) The demand for green products faced by the retailer is not only concerned with the retail price, but also influenced by the low-carbon preference of the consumer. The market demand function can be described by q=s−bp+λβ [14].

(2) The manufacturer has to improve its technology with a reduction cost of the carbon emission for reducing the carbon emission. This cost can be described by $C=1/2\cdot k\beta^{2}$ [41].

### 4.2. Centralized Pricing Decision-Making Model for LCSC

The LCSC is an idealized organization which aims to maximize the profits in the LCSC. Therefore, the manufacturer and the retailer face the sales market together. The wholesale price determined by the manufacturer is treated as an internal transfer price, which affects the profit of each member but does not affect the total profit. Thus, an appropriate retail price $p^{*}$ and a carbon emission reduction rate β* are set to maximize the profit function of the entire supply chain. The profit function can be expressed as

(1) $$\pi_{sc}^{*}=\left(p-c\right)\left(s-bp+\lambda \beta \right)+p_{c}\left[A-e\left(1-\beta \right)\left(s-bp+\lambda \beta \right)\right]-\frac{1}{2}k\beta^{2}$$

The hessian matrix $H_{1}(p,\beta )$ with respect to p and β can be expressed as

(2) $$H_{1}\left(p,\beta \right)=\left[\begin{array}{cc} -2b & \lambda -p_{c}eb\\ \lambda -p_{c}eb & 2p_{c}e\lambda -k \end{array}\right]$$

As the first order principal subformula $D_{1}=-2b<0$, if the second order principal subformula $D_{2}=2bk-{\left(\lambda +p_{c}eb\right)}^{2}>0$, $H_{1}(p,\beta )$ is a negative definite matrix. Let $\partial \pi_{sc}^{*}/\partial p=0$ and $\partial \pi_{sc}^{*}/\partial \beta =0$, then $p^{*}$ and $\beta^{*}$ in centralized pricing decision-making can be obtained:

(3) $$p^{*}=\frac{k\left(s+bc+p_{c}eb\right)-\left(\lambda +p_{c}eb\right)\left(p_{c}es+p_{c}e\lambda +c\lambda \right)}{2bk-{\left(\lambda +p_{c}eb\right)}^{2}}$$

(4) $$\beta^{*}=\frac{\left(s-bc-p_{c}eb\right)\left(\lambda +p_{c}eb\right)}{2bk-{\left(\lambda +p_{c}eb\right)}^{2}}$$

Next, we substitute $p^{*}$ and $\beta^{*}$ into the demand function q and the profit function of supply chain $\pi_{sc}^{*}$. Thus, $q^{*}$ and $\pi_{sc}^{*}$ in centralized pricing decision-making can be calculated:

(5) $$q^{*}=\frac{bk\left(s-bc-p_{c}eb\right)}{2bk-{\left(\lambda +p_{c}eb\right)}^{2}}$$

(6) $$\pi_{sc}^{*}=\frac{k{\left(s-bc-p_{c}eb\right)}^{2}}{2[2bk-\left(\lambda +p_{c}eb{)}^{2}\right]}+p_{c}A$$

If $2bk-{\left(\lambda +p_{c}eb\right)}^{2}>0$, the retail price and the carbon emission reduction rate are concave functions of the profit of the supply chain. Meanwhile, in order to ensure that they are non-negative, the condition of $k(s+bc+p_{c}eb)-(\lambda +p_{c}eb)(p_{c}es+p_{c}e\lambda +c\lambda )>0$ and $s-bc-p_{c}eb>0$ must be satisfied.

### 4.3. Decentralized Pricing Decision-Making Model for LCSC

In a decentralized pricing decision-making model, the manufacturer can be regarded as a Stackelberg leader who determines the optimal unit wholesale price of the product *w* and the carbon emission reduction rate *β*, and the retailer can be regarded as a follower who determines the retail price of the product *p*. Thus, the profit functions can be expressed as

(7) $$\pi_{m}^{n}=\left(w-c\right)\left(s-bp+\lambda \beta \right)+p_{c}\left[A-e\left(1-\beta \right)\left(s-bp+\lambda \beta \right)\right]-\frac{1}{2}k\beta^{2}$$

(8) $$\pi_{r}^{n}=\left(p-w\right)\left(s-bp+\lambda \beta \right)$$

By adopting the reverse derivation method, the retailer first determines p, and then the manufacturer determines *w* and β. Due to $\partial^{2}\pi_{r}^{n}/\partial p^{2}=-2b<0$, let $\partial \pi_{r}^{n}/\partial p=0$, then p determined by the retailer can be obtained as follows:

(9) $$p=\frac{s+\lambda \beta +bw}{2b}$$

The hessian matrix $H_{2}(w,\beta )$ with respect to the wholesale price w and the carbon emission reduction rate β can be expressed as follows:

(10) $$H_{2}\left(w,\beta \right)=\left[\begin{array}{cc} -b & \frac{\lambda -p_{c}eb}{2}\\ \frac{\lambda -p_{c}eb}{2} & p_{c}e\lambda -k \end{array}\right]$$

As the first order principal subformula $D_{1}=-b<0$, if the second order principal subformula $D_{2}=[4bk-{\left(\lambda +p_{c}eb\right)}^{2}]/4>0$, $H_{2}(w,\beta )$ is a negative definite matrix. Let $\partial \pi_{m}^{n}/\partial w=0$ and $\partial \pi_{m}^{n}/\partial \beta =0$, then $w^{n}$ and $\beta^{n}$ in decentralized pricing decision-making can be obtained as follows:

(11) $$w^{n}=\frac{2k\left(s+bc+p_{c}eb\right)-\left(\lambda +p_{c}eb\right)\left(p_{c}es+p_{c}e\lambda +c\lambda \right)}{4bk-{\left(\lambda +p_{c}eb\right)}^{2}}$$

(12) $$\beta^{n}=\frac{\left(s-bc-p_{c}eb\right)\left(\lambda +p_{c}eb\right)}{4bk-{\left(\lambda +p_{c}eb\right)}^{2}}$$

Next, we substitute $w^{n}$ and $\beta^{n}$ into the demand function q. Thus, $q^{n}$ and $p^{n}$ in decentralized pricing decision-making can be obtained as follows:

(13) $$q^{n}=\frac{bk\left(s-bc-p_{c}eb\right)}{4bk-{\left(\lambda +p_{c}eb\right)}^{2}}$$

(14) $$p^{n}=\frac{k\left(3s+bc+p_{c}eb\right)-\left(\lambda +p_{c}eb\right)\left(p_{c}es+p_{c}e\lambda +c\lambda \right)}{4bk-{\left(\lambda +p_{c}eb\right)}^{2}}$$

The optimal retailer profit $\pi_{r}^{n}$, the manufacturer profit $\pi_{m}^{n}$, and $\pi_{sc}^{n}$ in decentralized pricing decision-making can be calculated as follows:

(15) $$\pi_{r}^{n}=\frac{bk^{2}{\left(s-bc-p_{c}eb\right)}^{2}}{{\left[4bk-{\left(\lambda +p_{c}eb\right)}^{2}\right]}^{2}}$$

(16) $$\pi_{m}^{n}=\frac{k{\left(s-bc-p_{c}eb\right)}^{2}}{2[4bk-\left(\lambda +p_{c}eb{)}^{2}\right]}+p_{c}A$$

(17) $$\pi_{sc}^{n}=\pi_{r}^{n}+\pi_{m}^{n}=\frac{k{\left(s-bc-p_{c}eb\right)}^{2}(6bk-\left(\lambda +p_{c}eb{)}^{2}\right)}{2{\left[4bk-{\left(\lambda +p_{c}eb\right)}^{2}\right]}^{2}}+p_{c}A$$

Obviously, if $2bk-{\left(\lambda +p_{c}eb\right)}^{2}>0$, the second order principal subformula of the hessian matrix $H_{2}(w,\beta )$ is greater than zero. At the same time, if $s-bc-p_{c}eb>0$ and $k(s+bc+p_{c}eb)-(\lambda +p_{c}eb)(p_{c}es+p_{c}e\lambda +c\lambda )>0$, $w^{n}$, $\beta^{n}$ and $p^{n}$ are nonnegative.

### 4.4. Decentralized Pricing Decision-Making Model for LCSC

The manufacturer has the characteristic of fairness concern, while the retailer has the characteristic of fairness neutrality. As the decision-makers often only focus on the negative unfair utility to themselves, the manufacturer’s fairness concern coefficient $\eta_{m}$ is introduced by Du et al. [39]. Then, the utility functions can be expressed as

(18) $$U\left(\pi_{m}^{m}\right)=\pi_{m}-\eta_{m}\left(\pi_{r}-\pi_{m}\right)$$

(19) $$U\left(\pi_{r}^{m}\right)=\pi_{r}$$

The retailer and the manufacturer also follow the Stackelberg game when the manufacturer is concerned about the fairness. Similarly to Section 4.3, the retailer acts as a follower who maximizes their utility function based on the wholesale price and the carbon emission reduction rate set by the manufacturer.

Since $\partial^{2}U(\pi_{r}^{m})/\partial p^{2}=-2b<0$, let $\partial U(\pi_{r}^{m})/\partial p=0$; the optimal retail price p determined at the time of manufacturer’s fairness concern can be obtained as follows:

(20) $$p=\frac{s+\lambda \beta +bw}{2b}$$

Through substituting p into the manufacturer’s utility function $U(\pi_{m}^{m})$, the hessian matrix $H_{3}(w,\beta )$ and the carbon emission reduction rate β can be expressed as follows:

(21) $$H_{3}\left(w,\beta \right)=\left[\begin{array}{cc} \frac{-2b-3b\eta_{m}}{2} & \frac{\left(1+\eta_{m}\right)\left(\lambda -p_{c}eb\right)+\lambda \eta_{m}}{2}\\ \frac{\left(1+\eta_{m}\right)\left(\lambda -p_{c}eb\right)+\lambda \eta_{m}}{2} & \left(1+\eta_{m}\right)\left(p_{c}e\lambda -k\right)-\frac{\lambda^{2}\eta_{m}}{2b} \end{array}\right]$$

As the first order principal subformula $D_{1}=-(2b+3b\eta_{m})/2<0$, if the second order principal subformula $D_{2}=\{{\left(1+\eta_{m}\right)}^{2}[4bk-{\left(\lambda +p_{c}eb\right)}^{2}]+(1+\eta_{m})2bk\eta_{m}\}/4>0$, $H_{3}(w,\beta )$ is a negative definite matrix β. Let $\partial U(\pi_{m}^{m})/\partial w=0$ and $\partial U(\pi_{m}^{m})/\partial \beta =0$, the optimal wholesale price $w^{m}$ and the carbon emission reduction rate $\beta^{m}$ under manufacturer’s fairness concern can be obtained as follows:

(22) $$w^{m}=\frac{2k\left[\left(s+bc+p_{c}eb\right)\left(1+\eta_{m}\right)+s\eta_{m}\right]-\left(1+\eta_{m}\right)\left(\lambda +p_{c}eb\right)\left(p_{c}es+p_{c}e\lambda +c\lambda \right)}{\left(1+\eta_{m}\right)[4bk-\left(\lambda +p_{c}eb{)}^{2}\right]+2bk\eta_{m}}$$

(23) $$\beta^{m}=\frac{\left(s-bc-p_{c}eb\right)\left(\lambda +p_{c}eb\right)\left(1+\eta_{m}\right)}{\left(1+\eta_{m}\right)[4bk-\left(\lambda +p_{c}eb{)}^{2}\right]+2bk\eta_{m}}$$

Next, we substitute $w^{m}$ and $\beta^{m}$ into the demand function q and the utility function of manufacturer $U(\pi_{m}^{m})$. Thus, $q^{m}$ $p^{m}$ under manufacturer’s fairness concern can be obtained as follows:

(24) $$q^{m}=\frac{bk\left(s-bc-p_{c}eb\right)\left(1+\eta_{m}\right)}{\left(1+\eta_{m}\right)[4bk-\left(\lambda +p_{c}eb{)}^{2}\right]+2bk\eta_{m}}$$

(25) $$p^{m}=\frac{k\left[\left(3s+bc+p_{c}eb\right)\left(1+\eta_{m}\right)+2s\eta_{m}\right]-\left(1+\eta_{m}\right)\left(\lambda +p_{c}eb\right)\left(p_{c}es+p_{c}e\lambda +c\lambda \right)}{\left(1+\eta_{m}\right)[4bk-\left(\lambda +p_{c}eb{)}^{2}\right]+2bk\eta_{m}}$$

The optimal manufacturer profit $\pi_{m}^{m}$ and the profit $\pi_{sc}^{m}$ can be obtained as follows:

(26) $$\pi_{r}^{m}=\frac{bk^{2}{\left(s-bc-p_{c}eb\right)}^{2}{\left(1+\eta_{m}\right)}^{2}}{[\left(1+\eta_{m}\right){\left(4bk-\left(\lambda +p_{c}eb{)}^{2}\right)+2bk\eta_{m}\right]}^{2}}$$

(27) $$\pi_{m}^{m}=\frac{k{\left(s-bc-p_{c}eb\right)}^{2}\left(1+\eta_{m}\right)[\left(1+\eta_{m}\right)\left(4bk-\left(\lambda +p_{c}eb{)}^{2}\right)+4bk\eta_{m}\right]}{2[\left(1+\eta_{m}\right){\left(4bk-\left(\lambda +p_{c}eb{)}^{2}\right)+2bk\eta_{m}\right]}^{2}}+p_{c}A$$

(28) $$\pi_{sc}^{m}=\pi_{r}^{m}+\pi_{m}^{m}=\frac{k{\left(s-bc-p_{c}eb\right)}^{2}\left(1+\eta_{m}\right)[\left(1+\eta_{m}\right)\left(6bk-\left(\lambda +p_{c}eb{)}^{2}\right)+4bk\eta_{m}\right]}{2[\left(1+\eta_{m}\right){\left(4bk-\left(\lambda +p_{c}eb{)}^{2}\right)+2bk\eta_{m}\right]}^{2}}+p_{c}A$$

Similarly to Section 4.3, if $2bk-{\left(\lambda +p_{c}eb\right)}^{2}>0$, the second order principal subformula of the hessian matrix $H_{3}(w,\beta )$ is greater than zero. At the same time, if $s-bc-p_{c}eb>0$ and $k(s+bc+p_{c}eb)-(\lambda +p_{c}eb)(p_{c}es+p_{c}e\lambda +c\lambda )>0$, *w^m^*, $\beta^{m}$ and $p^{m}$ are nonnegative.

**Proposition** **1.** *In the decentralized pricing decision, the wholesale price with the manufacturer’s fairness concern is higher than the wholesale price with the manufacturer’s fairness neutrality, while the carbon emission reduction rate with the manufacturer’s fairness concern is lower. The carbon emission reduction rate in the decentralized pricing decision is lower than the rate in the centralized pricing decision.*

The proof of Proposition 1 is given in Appendix A.

Proposition 1 shows that if the manufacturer pays attention to fairness and wants to satisfy its own requirement to fairness, it will not only raise the wholesale price of the product, but also reduce the carbon emission reduction rate.

**Corollary** **1.** *In the decentralized pricing decision, the retail price with the manufacturer’s fairness concern is higher than the retail price with the manufacturer’s fairness neutrality, and the latter price is greater than the retail price in the centralized pricing decision. Similarly, in the decentralized pricing decision, the order quantity with the manufacturer’s fairness concern is lower than the order quantity with the manufacturer’s fairness neutrality, and the latter quantity is also lower than the order quantity in the centralized pricing decision.*

The proof of Corollary 1 is given in Appendix B.

Corollary 1 indicates that the manufacturer’s fairness concern forces the retailer to raise the retail price of the product and reduce the demand of the product.

**Corollary** **2.** *In the decentralized pricing decision, the profit of the supply chain in the case of the manufacturer’s fairness concern is smaller than the profit of the supply chain in the case of the manufacturer’s fairness neutrality, and the latter profit is smaller than the profit of the supply chain in the centralized pricing decision*.

The proof of Corollary 2 is given in Appendix C.

Corollary 2 shows that the manufacturer’s fair concern behavior not only reduces its own profit and that of the retailer, but also reduces the profit of the supply chain. This can be explained by the fact that the manufacturer pursues a fair profit distribution by sacrificing part of the profit to punish its rivals.

**Proposition** **2.** *In the decentralized pricing decision, the carbon emission reduction rate β^m^ and the order quantity $q^{m}$ are negatively correlated with the manufacturer’s fairness concern coefficient.*

The proof of Proposition 2 is given in Appendix D.

Proposition 2 shows that if manufacturer pays attention to fairness, the fairness concern coefficient has an influence on the decision variables.

**Proposition** **3.** *In the decentralized pricing decision, $\pi_{r}^{m}$ and $\pi_{m}^{m}$ are negatively correlated with $\eta_{m}$.*

The proof of Proposition 3 is given in Appendix E.

Proposition 3 shows that if manufacturer pays attention to fairness, the fairness concern coefficient has an influence on the profit of all members. This indicates that the manufacturer’s fairness concern coefficient not only hurts its own profit, but also hurts the profit of the downstream retailer.

### 4.5. Decentralized Pricing Decision-Making Model for LCSC Considering Retailer’s Fairness Concern

As the retailer has the characteristic of fairness concern and the manufacturer has the characteristic of fairness neutrality, the retailer is concerned with both its own profit and the fairness. By introducing the retailer’s fairness concern coefficient $\eta_{r}$ [39], the utility functions are expressed as follows:

(29) $$U\left(\pi_{m}^{r}\right)=\pi_{m}$$

(30) $$U\left(\pi_{r}^{r}\right)=\pi_{r}-\eta_{r}\left(\pi_{m}-\pi_{r}\right)$$

The retailer and the manufacturer follow the Stackelberg game where the retailer is concerned with the fairness. Similar to Section 4.4, the retailer first maximizes its utility function by using a reverse derivation. Since $\partial^{2}U(\pi r)/\partial p^{2}=-2b(1+\eta_{r})<0$, let $\partial U(\pi_{r}^{r})/\partial p=0$, then p determined by the retailer at the time of retailer’s fairness concern is obtained as follows:

(31) $$p=\frac{\left(1+\eta_{r}\right)\left(s+\lambda \beta \right)-\eta_{r}p_{c}eb\left(1-\beta \right)-b\left[\eta_{r}\left(c-2w\right)-w\right]}{2b\left(1+\eta_{r}\right)}$$

Through substituting p into the manufacturer’s utility function $U(\pi_{m}^{r})$, the hessian matrix $H_{4}(w,\beta )$ with respect to wholesale price w and β can be expressed:

(32) $$H_{4}\left(w,\beta \right)=\left[\begin{array}{cc} \frac{-b\left(2\eta_{r}+1\right)}{1+\eta_{r}} & \frac{\left(1+\eta_{r}\right)\left(\lambda -p_{c}eb\right)-2p_{c}eb\eta_{r}}{2\left(1+\eta_{r}\right)}\\ \frac{\left(1+\eta_{r}\right)\left(\lambda -p_{c}eb\right)-2p_{c}eb\eta_{r}}{2\left(1+\eta_{r}\right)} & \frac{\left(1+\eta_{r}\right)\left(p_{c}e\lambda -k\right)-p_{c}^{2}e^{2}b\eta_{r}}{1+\eta_{r}} \end{array}\right]$$

As the first order principal subformula $D_{1}=-b(2\eta_{r}+1)/(1+\eta_{r})<0$, if the second order principal subformula $D_{2}=\{(1+\eta_{r})[4bk-{\left(\lambda +p_{c}eb\right)}^{2}]+4bk\eta_{r}\}/4(1+\eta_{r})>0$, $H_{4}(w,\beta )$ is a negative definite matrix and $U(\pi_{m}^{r})$ is a concave function of w and β. Let $\partial U(\pi_{m}^{r})/\partial w=0$ and $\partial U(\pi_{m}^{r})/\partial \beta =0$, then $w^{r}$ and the carbon emission reduction rate $\beta^{r}$ under retailer’s fairness concern can be obtained as follows:

(33) $$w^{r}=\frac{2k\left[\left(s+bc+p_{c}eb\right)\left(1+\eta_{r}\right)+2\eta_{r}\left(p_{c}eb+bc\right)\right]-\left(1+\eta_{r}\right)\left(\lambda +p_{c}eb\right)\left(p_{c}es+p_{c}e\lambda +c\lambda \right)}{\left(1+\eta_{r}\right)[4bk-\left(\lambda +p_{c}eb{)}^{2}\right]+4bk\eta_{r}}$$

(34) $$\beta^{r}=\frac{\left(s-bc-p_{c}eb\right)\left(\lambda +p_{c}eb\right)\left(1+\eta_{r}\right)}{\left(1+\eta_{r}\right)[4bk-\left(\lambda +p_{c}eb{)}^{2}\right]+4bk\eta_{r}}$$

Next, we substitute $w^{r}$ and $\beta^{r}$ into the demand function q and the utility function of manufacturer $U(\pi_{m}^{r})$. Thus, $q^{r}$ and $p^{r}$ under retailer’s fairness concern can be obtained as follows:

(35) $$q^{r}=\frac{bk\left(s-bc-p_{c}eb\right)\left(1+3\eta_{r}\right)}{\left(1+\eta_{r}\right)[4bk-\left(\lambda +p_{c}eb{)}^{2}\right]+4bk\eta_{r}}$$

(36) $$p^{r}=\frac{k\left[\left(3s+bc+p_{c}eb\right)\left(1+\eta_{r}\right)+2\eta_{r}\left(s+bc+p_{c}eb\right)\right]-\left(1+\eta_{r}\right)\left(\lambda +p_{c}eb\right)\left(p_{c}es+p_{c}e\lambda +c\lambda \right)}{\left(1+\eta_{r}\right)[4bk-\left(\lambda +p_{c}eb{)}^{2}\right]+4bk\eta_{r}}$$

Through substituting $w^{r}$, $\beta^{r}$ and $p^{r}$ into the manufacturer’s utility function and the retailer’s utility function, the profit can be obtained as follows, respectively:

(37) $$\pi_{r}^{r}=\frac{bk^{2}{\left(s-bc-p_{c}eb\right)}^{2}{\left(1+3\eta_{r}\right)}^{2}}{[\left(1+\eta_{r}\right){\left(4bk-\left(\lambda +p_{c}eb{)}^{2}\right)+4bk\eta_{r}\right]}^{2}}$$

(38) $$\pi_{m}^{r}=\frac{k{\left(s-bc-p_{c}eb\right)}^{2}\left(1+\eta_{r}\right)[\left(1+\eta_{r}\right)\left(4bk-\left(\lambda +p_{c}eb{)}^{2}\right)+8bk\eta_{r}\right]}{2[\left(1+\eta_{r}\right){\left(4bk-\left(\lambda +p_{c}eb{)}^{2}\right)+4bk\eta_{r}\right]}^{2}}+p_{c}A$$

(39) $$\pi_{sc}^{r}=\pi_{r}^{r}+\pi_{m}^{r}=\frac{k{\left(s-bc-p_{c}eb\right)}^{2}[{\left(1+\eta_{r}\right)}^{2}\left(6bk-\left(\lambda +p_{c}eb{)}^{2}\right)+24bk\eta_{r}^{2}+16bk\eta_{r}\right]}{2[\left(1+\eta_{r}\right){\left(4bk-\left(\lambda +p_{c}eb{)}^{2}\right)+4bk\eta_{r}\right]}^{2}}+p_{c}A$$

Similarly to Section 4.3, if $2bk-{\left(\lambda +p_{c}eb\right)}^{2}>0$, the second order principal subformula of the hessian matrix $H_{4}(w,\beta )$ is greater than zero. At the same time, if $s-bc-p_{c}eb>0$ and $k(s+bc+p_{c}eb)-(\lambda +p_{c}eb)(p_{c}es+p_{c}e\lambda +c\lambda )>0$, $w^{r}$, $\beta^{r}$ and $p^{r}$ are nonnegative.

**Proposition** **4.** *The wholesale price is lower than those under the fairness neutrality in the pricing decision.*

The proof of Proposition 4 is given in Appendix F.

Proposition 4 indicates that if the retailer pays attention to fairness, the manufacturer decreases the wholesale price and its carbon emission reduction rates.

**Corollary** **3.** *(i) The retail price in the decentralized pricing decision with the manufacturer’s fairness concern is lower than the retail price in the decentralized pricing decision with fairness neutrality. (ii) If $\lambda \leq p_{c}eb$, the retail price in the decentralized pricing decision with the manufacturer’s fairness concern is higher than the retail price in the centralized pricing decision. (iii) When $\lambda >p_{c}eb$, let $(\lambda^{2}+p_{c}eb\lambda -bk)/(\lambda^{2}-p_{c}^{2}e^{2}b^{2})=H$. If $H<-\eta_{r}/(\eta_{r}+1)$, the retail price in the decentralized pricing decision is higher than the retail price in the centralized pricing decision, and if $-\eta_{r}/(\eta_{r}+1)<H<1/2$, the latter retail price is higher than the former retail price.*

The proof of Corollary 3 is given in Appendix G.

Corollary 3 shows that if retailer cares about fairness, they will reduce the retail price to increase the sales volume.

**Corollary** **4.** *(i) The profit of the retailer in the decentralized pricing decision with fairness concern is larger than the profit of the retailer in the decentralized pricing decision with fairness neutrality, while the profit of the manufacturer in the decentralized pricing decision with fairness concern is smaller than the profit of the manufacturer in the decentralized pricing decision with fairness neutrality. (ii) Let $[4bk-{\left(\lambda +p_{c}eb\right)}^{2}][2bk-{\left(\lambda +p_{c}eb\right)}^{2}]/(4b^{2}k^{2})=G$. If $G\geq \eta_{r}/(2\eta_{r}+1)$, the profit in the decentralized pricing decision is larger than the profit obtained in the decentralized pricing decision with fairness neutrality. If $G<\eta_{r}/(2\eta_{r}+1)$, the former profit is smaller than the latter profit. (iii) The profit of the supply chain in the decentralized pricing decision with the manufacturer’s fairness is smaller than the profit in the the centralized pricing decision.*

The proof of Corollary 4 is given in Appendix H.

Corollary 4 shows that the retailer’s fairness concern coefficient increases the profit of the retailer and decreases the profit of the manufacturer, and the profit of the supply chain in the decentralized pricing decision is smaller than the profit of the supply chain in the centralized pricing decision. This can be explained by the fact that the retailer pursues the fair profit allocation, which sacrifices the profit of its rival to increase its own profit.

**Proposition** **5.** *In the decentralized pricing decision, $\beta^{r}$, $w^{r}$ and $p^{r}$ are negatively correlated with the retailer’s fairness concern coefficient $\eta_{r}$, and the order quantity $q^{r}$ is positively correlated with $\eta_{r}$.*

The proof of Proposition 5 is given in Appendix I.

Proposition 5 indicates that if the retailer pays attention to fairness, the fairness concern coefficient has an influence on the decision variables.

**Proposition** **6.** *In the decentralized pricing decision with the retailer’s fairness concern, $\pi_{r}^{r}$ is positively correlated with $\eta_{r}$.*

The proof of Proposition 6 is given in Appendix J.

Proposition 6 indicates that if the retailer pays attention to fairness, the fairness concern coefficient has an effect on the profits of the members. Specifically, the retailer’s profit increases with the increase of the retailer’s fairness concern coefficient.

## 5. Numerical Analysis

Similar to Yang et al. [42], we give some estimated parameters as follows: *s* = 300,, b=5, c=4, k=600, $p_{c}=1$, e=2, λ=1 and A=500.

### 5.1. Analysis of the Impact of the Fairness Concern of the Manufacturer and the Retailer on Pricing Decisions

**Figure 2:**
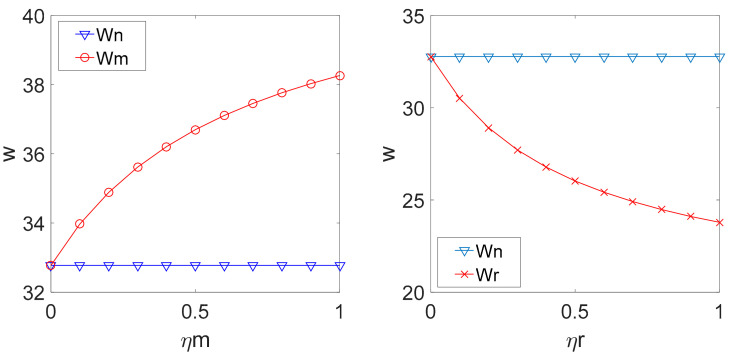
Relationship among $\eta_{m}$, $\eta_{r}$ and w.

By substituting the above parameters into Equation (4), (11), (12), (22), (23), (33) and (34), the relationship among w, β, and $\eta_{m}$, $\eta_{r}$ in different decision models can be shown in Figure 1 and Figure 2.

Figure 2 shows that if the manufacturer pays attention to fairness, the wholesale price in the decentralized LCSC decision with fairness concern is higher than that in the decentralized LCSC decision with fairness neutrality and goes up with an increasing manufacturer’s fairness concern coefficient. If the retailer pays attention to fairness, the wholesale price in the decentralized LCSC decision with fairness concern is lower than that in the decentralized LCSC decision with fairness neutrality and goes down when the retailer’s fairness concern coefficient increases. If the manufacturer is fair-minded, the manufacturer will increase the wholesale price compensate for the cost of emission reduction. If the retailer is fair-minded, the manufacturer will decrease the wholesale price to obtain a larger profit.

**Figure 3:**
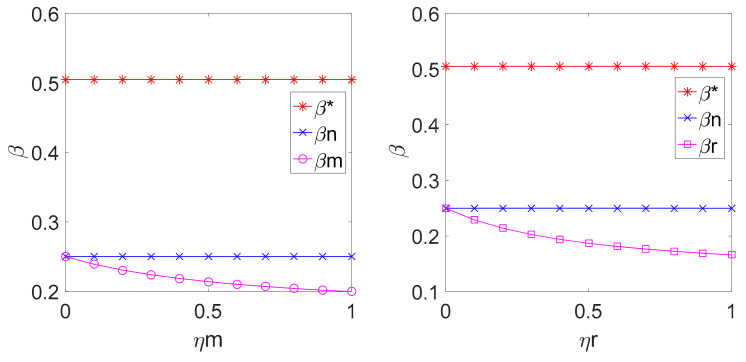
Relationship among $\eta_{m}$, $\eta_{r}$ and β.

Figure 3 suggests that if the manufacturer or the retailer has fairness concern in the decentralized LCSC decision, the carbon emission reduction rate is smaller than that in the decentralized LCSC decision with fairness neutrality, and the latter is smaller than that in the centralized LCSC decision. If the manufacturer pays attention to fairness, the manufacturer is going to reduce the emission reduction rate to lower the cost. If the retailer is concerned with fairness, the profit allocation inclines to the retailer with the increasing of fairness concern and the manufacturer must reduce the carbon emission rate to reduce costs.

**Figure 4:**
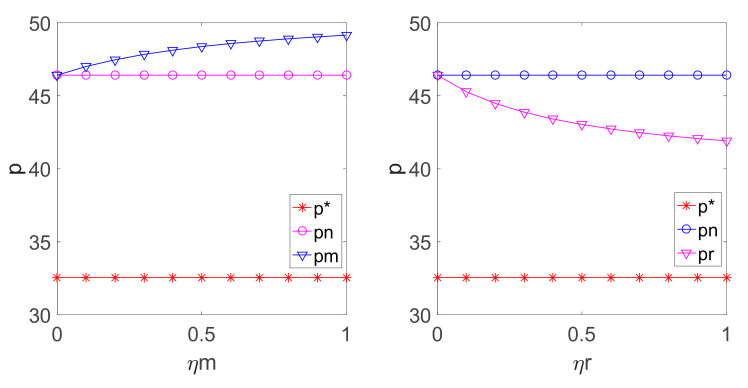
Relationship among $\eta_{m}$, $\eta_{r}$ and p.

By substituting the above parameters into Equations (3), (5), (13), (14), (24), (25), (35) and (36), the relationship among p, q, and the fairness concern coefficient $\eta_{m}$, $\eta_{r}$ in different decision-making models can be obtained as shown in Figure 3 and Figure 4.

**Figure 5:**
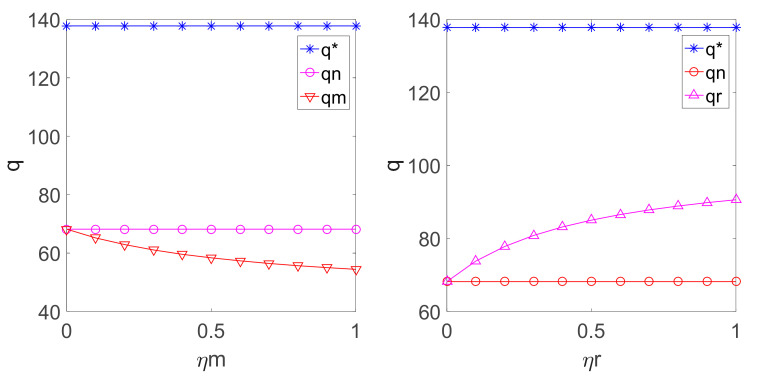
Relationship among $\eta_{m}$, $\eta_{r}$ and q.

According to Figure 4 and Figure 5, it can be seen that if the manufacturer is fair-minded, the retail price will be greater than that with fairness neutrality and increases when the manufacturer’s fairness concern coefficient increases. At the same time, if the retailer pays attention to fairness, the retail price will be lower than that with fairness neutrality. Moreover, the order quantity increases as the manufacturer’s fairness concern coefficient increases. This means that if manufacturer is concerned with fairness, the profit allocation will be inclined to the manufacturer, and the retailer will raise the retail price to get more profit, which can reduce the demand.

### 5.2. Analysis of the Impact of the Fairness Concern of the Manufacturer and the Retailer on the Profit of the Supply Chain

**Figure 6:**
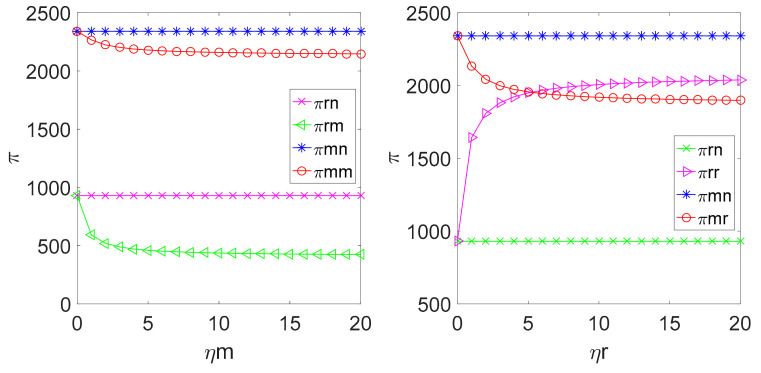
Relationship among $\eta_{m}$ and $\eta_{r}$.

**Figure 7:**
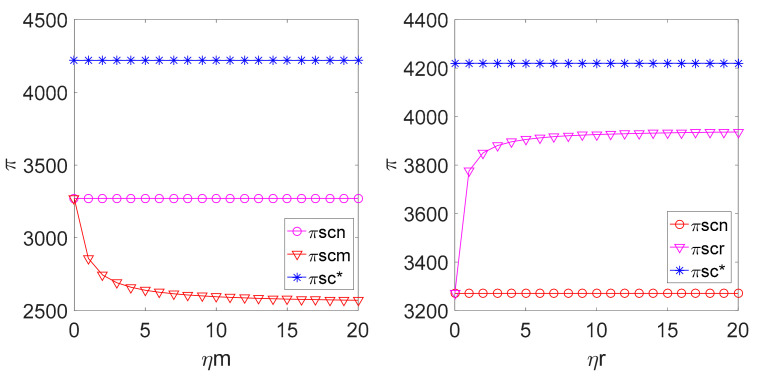
Relationship among $\eta_{m}$, $\eta_{r}$ and $\pi_{sc}$.

By replacing the above parameters into the profit expressions of the retailer, the manufacturer and the whole LCSC under different decision modes, the relationship among $\pi_{r}$, $\pi_{m}$, $\pi_{sc}$ and the fairness concern coefficient $\eta_{m}$, $\eta_{r}$ in different decision-making models can be obtained as shown in Figure 6 and Figure 7.

Figure 6 shows that if the manufacturer is fair-minded, the profit of both the manufacturer and the retailer is lower than their corresponding profits with fairness neutrality. Simultaneously, the retailer’s profit is smaller. The retailer’s profit is higher than its profit in the case of fairness neutrality. In addition, if the fairness concern coefficient $\eta_{r}$ reaches a certain value, the retailer’s profit is going to be greater than the manufacturer’s profit. Figure 6 illustrates that the fairness concern coefficient $\eta_{m}$ reduces the manufacturer’s profit and the retailer’s profit, which means that the manufacturer pursues fairness by sacrificing part of its profit to punish the retailer; $\eta_{r}$ means that the retailer makes the profit allocation incline to itself.

As shown in Figure 7, the profit of the supply chain in the case of the manufacturer’s fairness concern is lower than the profit generated in the case of the manufacturer’s fairness neutrality, which is also lower than the profit obtained in the case of centralized pricing decision-making and in the case of the retailer’s fairness concern, and if the consumers’ low carbon preference is lower, the profit of supply chain is higher than the profit generated in the case of fairness neutrality and lower than the profit generated in the case of centralized decision-making. This suggests that the nature of fairness concern undermines the profit of the supply chain. It can be understood that when the members of the supply chain are concerned about fairness, they will pay more attention to the fairness of profit allocation not the maximum profit in pricing decision-making.

### 5.3. Analysis of the Impact of the Consumer’s Low Carbon Preference on the Profit of the Supply Chain

**Figure 8:**
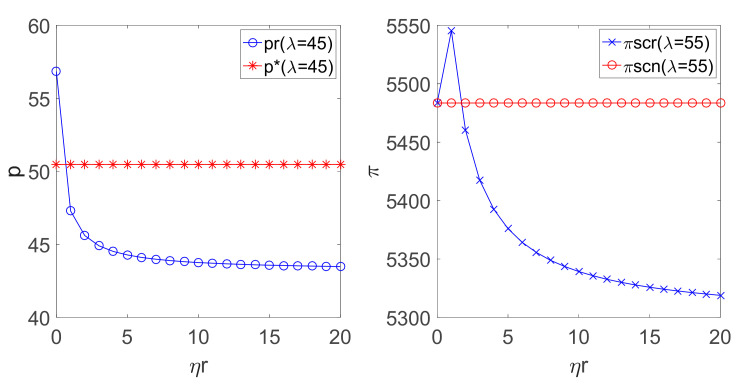
Relationship among p, $\pi_{sc}$ and $\eta_{r}$.

To verify Corollary 3 and Corollary 4, we increase the consumer’s low carbon preference coefficient by setting λ=45 (H<0) and λ=55 (G<1/2), respectively. Then, the relationship among the retail price p, the supply chain system profit $\pi_{sc}$ and the fairness concern coefficient $\eta_{r}$ can be obtained by substituting the above parameters into the expressions of the sales price and the profit in different decision modes, which are illustrated as Figure 8.

As can be seen from Figure 8, in the case of the consumer possessing a large low-carbon preference coefficient, if the retailer cares about fairness, the retail price will decrease as the fairness concern coefficient $\eta_{r}$ increases. When $\eta_{r}$ reaches a certain value, the retail price in the decentralized pricing decision with fairness concern is smaller than that in the centralized LCSC decision. When $\eta_{r}$ reaches a certain value, the total profit of the LCSC in the decentralized decision with the fairness concern is lower than that in the decentralized decision with fairness neutrality. This means that if consumers have a high preference for low carbon, the manufacturer needs to invest more in the cost of reducing carbon emissions, and when $\eta_{r}$ increases, if the retailer pursues more fairness, the manufacturer’s profit will be decreased more. Thus, both the incentive of the manufacturer for carbon emissions and the demand of the product will be reduced. Therefore, the retailer has to decrease the sales price to gain more profit, which reduces the profit of the supply chain finally.

In summary, this paper analyzes the impact of the fairness concerns of the supply chain members and the consumers’ low-carbon preferences on supply chain pricing decisions with the carbon quota policy. The research results show that the fairness concerns of members in a supply chain and the low-carbon preferences of consumers have a significant impact on the pricing decisions and sustainability levels of the supply chain. Among them, the fairness concern behavior of the manufacturer harms its own benefit, and the fairness concern behavior of the retailer improves its negotiation ability. Although the manufacturer increased costs due to emission reduction technologies, it is not suitable to consider fairness concerns. The retailer should also control the degree of fairness concerns, otherwise it will reduce the enthusiasm of the manufacturer. Compared with previous studies like Liu et al. (2017), we investigated an optimal pricing decision problem in a low-carbon supply chain from a new perspective. The results of this paper can contribute to understanding the relationship among fairness concern, retail price of the product and carbon emission reduction rate. Thus, this study can help decision-makers of the low-carbon supply chain to select optimal low-carbon and price strategies, which is beneficial to the sustainability of the supply chain.

## 6. Conclusions

This research studies a LCSC decision-making problem with fairness concern by considering the carbon quota policy. The LCSC includes a manufacturer and a retailer, in which the government gives a free quota of carbon to the manufacturer who applies low-carbon technologies to reduce its emission. The effects of the fairness concern coefficient on the players’ decisions are discussed in the paper.

For the studied LCSC, our results show that the total profit of the LCSC under the centralized decision are higher than that under the decentralized decision when fairness is considered. If the manufacturer is concerned with fairness, the fairness concern coefficient further reduces the carbon emission reduction rate and the LCSC’s total profit. If the retailer is concerned with fairness and the consumers have low demand for low-carbon products, the fairness concern coefficient will further boost the LCSC’s total profit. Meanwhile, if the consumers have high preferences for low-carbon products, the fairness concern coefficient of the retailer would result in a lower retail price compared with the retail price of centralized pricing decision-making, and the LCSC’s total profit will be further reduced.

Note that our study still has some limitations. First of all, this paper does not consider the double-fairness concern characteristic, in which the manufacturer and the retailer may have fairness concern at the same time. Secondly, the coordination of a low-carbon supply chain achieved by contract can be explored. Finally, cooperative emission reduction may be conducive to improve the sustainable development level of the supply chain. These provide the directions for our future study.

## Figures and Tables

**Table 1:** Parameters and decision variables.

**Parameters**	
c	manufacturer’s unit production cost
q	green product demand function
e	unit initial carbon emissions from the manufacturer
A	free carbon quotas provided by the government
s	total volume of the sales market
b	sensitivity coefficient of retail sale price, where s−bp>0
λ	low carbon preference coefficient of consumer
k	cost coefficient of carbon reduction investment
$p_{c}$	unit carbon price
$\eta_{r}$	fairness concern coefficient of the retailer, where $\eta_{r}>0$
$\eta_{m}$	fairness concern coefficient of the manufacturer, where $\eta_{m}>0$
$\pi_{r}$	the retailer’s profit function
$\pi_{m}$	the manufacturer’s profit function
$\pi_{sc}$	profit function of the supply chain
$U(\pi_{r})$	the retailer’s utility function
$U(\pi_{m})$	the manufacturer’s utility function
$U(\pi_{sc})$	utility function of the supply chain
${\left(\;\right)}^{*}$	centralized policy
${\left(\;\right)}^{n}$	decentralized policy
${\left(\;\right)}^{m}$	manufacturer’s fairness concern policy
${\left(\;\right)}^{r}$	retailer’s fairness concern policy
**Decision variables**	
w	unit wholesale price of the green product determined by the manufacturer
p	unit retail price of the green product determined by the retailer
β	carbon emission reduction rate per unit of green product

## Data Availability

Not applicable.

## Appendix A

**Proof of Proposition** **1.** By comparing $w^{n}$, $w^{m}$ and $p^{n}$, we can get $w^{n}-w^{m}=\frac{2bk\eta_{m}(w^{n}-s/b)}{(1+\eta_{m})[4bk-{\left(\lambda +p_{c}eb\right)}^{2}]+2bk\eta_{m}}$, $p^{n}=\frac{k(s-bc-p_{c}eb)}{4bk-{\left(\lambda +p_{c}eb\right)}^{2}}+w^{n}$. It can be concluded that w and p are feasible and nonnegative under the following conditions,$2bk-{\left(\lambda +p_{c}eb\right)}^{2}>0$, $k(s+bc+p_{c}eb)-(\lambda +p_{c}eb)(p_{c}es+p_{c}e\lambda +c\lambda )>0$ and $s-bc-p_{c}eb>0$. Therefore, it is easy to get $w^{n}<p^{n}$. According to Hypothesis 1, we have $w^{n}-s/b<p^{n}-s/b<0$ and $w^{n}<w^{m}$. By comparing $\beta^{n}$, $\beta^{*}$ and $\beta^{m}$, we can get $\frac{\beta^{n}}{\beta^{*}}=\frac{2bk-{\left(\lambda +p_{c}eb\right)}^{2}}{4bk-{\left(\lambda +p_{c}eb\right)}^{2}}<1$, $\frac{\beta^{m}}{\beta^{n}}=\frac{\left(1+\eta_{m})[4bk-{\left(\lambda +p_{c}eb\right)}^{2}\right]}{(1+\eta_{m})[4bk-{\left(\lambda +p_{c}eb\right)}^{2}]+2bk\eta_{m}}<1$. Thus, we have $\beta^{m}<\beta^{n}<\beta^{*}$. Proposition 1 is demonstrated. □

## Appendix B

**Proof of Corollary** **1.** By comparing $p^{n}$, $p^{m}$ and $p^{*}$, we can get $p^{*}-p^{n}=\frac{2bk(p^{*}-s/b)}{4bk-{\left(\lambda +p_{c}eb\right)}^{2}}$, $p^{n}-p^{m}=\frac{2bk\eta_{m}(p^{n}-s/b)}{(1+\eta_{m})[4bk-{\left(\lambda +p_{c}eb\right)}^{2}]+2bk\eta_{m}}$ Similar to proposition 1, we know that $p^{*}-p^{n}<0$ and $p^{n}-p^{m}<0$. Thus, we have $p^{*}<p^{n}<p^{m}$. By comparing formulas $q^{n}$, $q^{m}$ and $q^{*}$, we can get $\frac{q^{n}}{q^{*}}=\frac{2bk-{\left(\lambda +p_{c}eb\right)}^{2}}{4bk-{\left(\lambda +p_{c}eb\right)}^{2}}<1$, $\frac{q^{m}}{q^{n}}=\frac{\left(1+\eta_{m})[4bk-{\left(\lambda +p_{c}eb\right)}^{2}\right]}{(1+\eta_{m})[4bk-{\left(\lambda +p_{c}eb\right)}^{2}]+2bk\eta_{m}}<1$. Thus, we have $q^{m}<q^{n}<q^{*}$. Corollary 1 is demonstrated. □

## Appendix C

**Proof of Corollary** **2.** By comparing $\pi_{sc}^{n}$,$\pi_{sc}^{m}$ and $\pi_{sc}^{*}$ we obtain $\pi_{sc}^{n}-\pi_{sc}^{m}=\frac{2b^{2}k^{3}\eta_{m}{\left(s-bc-p_{c}eb\right)}^{2}[(3\eta_{m}+2)(4bk-{\left(\lambda +p_{c}eb\right)}^{2})+2bk\eta_{m}]}{{\left[4bk-{\left(\lambda +p_{c}eb\right)}^{2}\right]}^{2}{\left[(\eta_{m}+1)(4bk-{\left(\lambda +p_{c}eb\right)}^{2})+2bk\eta_{m}\right]}^{2}}$, $\pi_{sc}^{*}-\pi_{sc}^{n}=\frac{2b^{2}k^{3}{\left(s-bc-p_{c}eb\right)}^{2}}{[2bk-{\left(\lambda +p_{c}eb\right)}^{2}]{\left[4bk-{\left(\lambda +p_{c}eb\right)}^{2}\right]}^{2}}$. Since $2bk-{\left(\lambda +p_{c}eb\right)}^{2}>0$ and $s-bc-p_{c}eb>0$, we have $\pi_{\mathrm{sc}}^{n}<\pi_{\mathrm{sc}}^{n}<\pi_{\mathrm{sc}}^{*}$. By comparing $\pi_{r}^{n}$ and $\pi_{r}^{m}$, $\pi_{m}^{n}$ and $\pi_{m}^{m}$, respectively, we can get $\frac{\pi_{r}^{m}}{\pi_{r}^{n}}=\frac{{\left[(1+\eta_{m})(4bk-{\left(\lambda +p_{c}eb\right)}^{2})\right]}^{2}}{{\left[(1+\eta_{m})(4bk-{\left(\lambda +p_{c}eb\right)}^{2})+2bk\eta_{m}\right]}^{2}}<1$, $\frac{\pi_{m}^{m}-p_{c}A}{\pi_{m}^{n}-p_{c}A}=\frac{\left[(1+\eta_{m})(4bk-{\left(\lambda +p_{c}eb\right)}^{2})+4bk\eta_{m}][(1+\eta_{m})(4bk-{\left(\lambda +p_{c}eb\right)}^{2})\right]}{{\left[(1+\eta_{m})(4bk-{\left(\lambda +p_{c}eb\right)}^{2})+2bk\eta_{m}\right]}^{2}}<1$. Thus, we have $\pi_{r}^{m}<\pi_{r}^{n}$ and $\pi_{m}^{m}<\pi_{m}^{n}$. Corollary 2 is demonstrated. □

## Appendix D

**Proof of Proposition** **2.** Since $\beta^{m}$, $q^{m}$, $w^{m}$ and $p^{m}$ are derived from $\eta_{m}$ respectively, we can get

$$\begin{array}{cc} \frac{\delta \beta^{m}}{\delta \eta_{m}}=\frac{-2bk(\lambda +p_{c}eb)(s-bc-p_{c}eb)}{{\left[(\eta_{m}+1)(4bk-{\left(\lambda +p_{c}eb\right)}^{2})+2bk\eta_{m}\right]}^{2}} & \frac{\delta q^{m}}{\delta \eta_{m}}=\frac{-2b^{2}k^{2}(s-bc-p_{c}eb)}{{\left[(\eta_{m}+1)(4bk-{\left(\lambda +p_{c}eb\right)}^{2})+2bk\eta_{m}\right]}^{2}}\\ \frac{\delta w^{m}}{\delta \eta_{m}}=\frac{-2bk[4bk-{\left(\lambda +p_{c}eb\right)}^{2}][w^{n}-s/b]}{{\left[(\eta_{m}+1)(4bk-{\left(\lambda +p_{c}eb\right)}^{2})+2bk\eta_{m}\right]}^{2}} & \frac{\delta p^{m}}{\delta \eta_{m}}=\frac{-2bk[4bk-{\left(\lambda +p_{c}eb\right)}^{2}][p^{n}-s/b]}{{\left[(\eta_{m}+1)(4bk-{\left(\lambda +p_{c}eb\right)}^{2})+2bk\eta_{m}\right]}^{2}} \end{array}$$

Since $2bk-{\left(\lambda +p_{c}eb\right)}^{2}>0$ and $s-bc-p_{c}eb>0$, we have $\delta \beta^{m}/\delta \eta_{m}<0$ and $\delta q^{m}/\delta \eta_{m}<0$. Similar to proposition 1, it is easy to know $w^{n}-s/b<p^{n}-s/b<0$, then we have $\delta w^{m}/\delta \eta_{m}>0$ and $\delta p^{m}/\delta \eta_{m}>0$. Proposition 2 is demonstrated. □

## Appendix E

**Proof of Proposition** **3.** Since $\pi_{r}^{m}$ and $\pi_{m}^{m}$ are derived from $\eta_{m}$ respectively, we can get

$$\begin{array}{cc} \frac{\delta \pi_{r}^{m}}{\delta \eta_{m}}=\frac{-4b^{2}k^{3}(\eta_{m}+1){\left(s-bc-p_{c}eb\right)}^{2}}{{\left[(\eta_{m}+1)(4bk-{\left(\lambda +p_{c}eb\right)}^{2})+2bk\eta_{m}\right]}^{3}} & \frac{\delta \pi_{m}^{m}}{\delta \eta_{m}}=\frac{-4b^{2}k^{3}\eta_{m}{\left(s-bc-p_{c}eb\right)}^{2}}{{\left[(\eta_{m}+1)(4bk-{\left(\lambda +p_{c}eb\right)}^{2})+2bk\eta_{m}\right]}^{3}} \end{array}$$

Since $2bk-{\left(\lambda +p_{c}eb\right)}^{2}>0$ and $s-bc-p_{c}eb>0$, we have $\delta \pi_{r}^{m}/\delta \eta_{m}<0$ and $\delta \pi_{m}^{m}/\delta \eta_{m}<0$. Proposition 3 is demonstrated. □

## Appendix F

**Proof of Proposition** **4.** By comparing $w^{n}$ and $w^{r}$, $\beta^{n}$ and $\beta^{r}$, we can get

$$w^{n}-w^{r}=\frac{4k\eta_{r}(s-bc-p_{c}eb)[2bk-p_{c}eb(\lambda +p_{c}eb)]}{\left[4bk-{\left(\lambda +p_{c}eb\right)}^{2}][(1+\eta_{r})(4bk-{\left(\lambda +p_{c}eb\right)}^{2})+4bk\eta_{r}\right]}$$

$$\frac{\beta^{r}}{\beta^{n}}=\frac{\left(\eta_{r}+1)[4bk-{\left(\lambda +p_{c}eb\right)}^{2}\right]}{(\eta_{r}+1)[4bk-{\left(\lambda +p_{c}eb\right)}^{2}]+4bk\eta_{r}}$$

Since $2bk-{\left(\lambda +p_{c}eb\right)}^{2}>0$ and $s-bc-p_{c}eb>0$, we have *w^r^* < *w^n^* and $\beta^{r}<\beta^{n}$. Proposition 4 is demonstrated. □

## Appendix G

**Proof of Corollary** **3.** (i) By comparing $p^{n}$ and $p^{r}$, we can get

$$p^{n}-p^{r}=\frac{2k\eta_{r}(s-bc-p_{c}eb)[2bk-(\lambda +p_{c}eb)(\lambda -p_{c}eb)]}{\left[4bk-{\left(\lambda +p_{c}eb\right)}^{2}][(\eta_{r}+1)(4bk-{\left(\lambda +p_{c}eb\right)}^{2})+4bk\eta_{r}\right]}$$

Since $2bk-{\left(\lambda +p_{c}eb\right)}^{2}>0$ and $s-bc-p_{c}eb>0$, we have *p^n^* > *p^r^*. (ii) By comparing $p^{*}$ and $p^{r}$, we can get $p^{*}-p^{r}=\frac{2k(s-bc-p_{c}eb)[(\eta_{r}+1)(\lambda^{2}+p_{c}eb\lambda -bk)+\eta_{r}(\lambda^{2}-p_{c}^{2}e^{2}b^{2})]}{\left[2bk-{\left(\lambda +p_{c}eb\right)}^{2}][(\eta_{r}+1)(4bk-{\left(\lambda +p_{c}eb\right)}^{2})+4bk\eta_{r}\right]}$. If $\lambda =p_{c}eb$, we have $\lambda^{2}+p_{c}eb\lambda -bk=2\lambda^{2}-bk$. Since $2bk-{\left(\lambda +p_{c}eb\right)}^{2}>0$, we have $2\lambda^{2}-bk<0$. Furthermore, since $s-bc-p_{c}eb>0$, we have *p** < *p^r^*. If $\lambda <p_{c}eb$, let $(\lambda^{2}+p_{c}eb\lambda -bk)/(\lambda^{2}-p_{c}^{2}e^{2}b^{2})=H$, we have $(1-2H)(\lambda^{2}-p_{c}^{2}e^{2}b^{2})=2bk-{\left(\lambda +p_{c}eb\right)}^{2}>0$. Then, it is easy to get H>1/2. Based on the expression of H, we have $\lambda^{2}+p_{c}eb\lambda -bk<0$. Furthermore, since $s-bc-p_{c}eb>0$, we have *p** < *p^r^*. (iii) If $\lambda >p_{c}eb$, let $(\lambda^{2}+p_{c}eb\lambda -bk)/(\lambda^{2}-p_{c}^{2}e^{2}b^{2})=H$, we have $(1-2H)(\lambda^{2}-p_{c}^{2}e^{2}b^{2})=2bk-{\left(\lambda +p_{c}eb\right)}^{2}>0$. Then, it is easy to get H<1/2. Let $(1+\eta_{r})H+\eta_{r}>0$, i.e., $-\eta_{r}/(\eta_{r}+1)<H<1/2$, then we have *p^r^* < *p**. Let $(1+\eta_{r})H+\eta_{r}<0$, i.e., $H<-\eta_{r}/(\eta_{r}+1)$, we have *p^r^* > *p**. Corollary 3 is demonstrated. □

## Appendix H

**Proof of Corollary** **4.** (i) By comparing $\pi_{r}^{r}$ and $\pi_{r}^{n}$, $\pi_{m}^{r}$ and $\pi_{m}^{n}$ respectively, we can get

$$\frac{\pi_{r}^{r}}{\pi_{r}^{n}}=\frac{{\left[(\eta_{r}+1)(4bk-{\left(\lambda +p_{c}eb\right)}^{2})+2\eta_{r}(4bk-{\left(\lambda +p_{c}eb\right)}^{2})\right]}^{2}}{{\left[(\eta_{r}+1)(4bk-{\left(\lambda +p_{c}eb\right)}^{2})+4bk\eta_{r}\right]}^{2}}$$

$$\frac{\pi_{m}^{r}-p_{c}A}{\pi_{m}^{n}-p_{c}A}=\frac{{\left[(1+\eta_{r})(4bk-{\left(\lambda +p_{c}eb\right)}^{2})\right]}^{2}+8bk\eta_{r}(1+\eta_{r})[4bk-{\left(\lambda +p_{c}eb\right)}^{2}]}{{\left[(1+\eta_{r})(4bk-{\left(\lambda +p_{c}eb\right)}^{2})+4bk\eta_{r}\right]}^{2}}<1$$

Since $2\eta_{r}[4bk-{\left(\lambda +p_{c}eb\right)}^{2}]-4bk\eta_{r}=2\eta_{r}[2bk-{\left(\lambda +p_{c}eb\right)}^{2}]>0$, we have $\pi_{r}^{r}>\pi_{r}^{n}$ and $\pi_{m}^{r}<\pi_{m}^{n}$. (ii) By comparing $\pi_{sc}^{r}$ and $\pi_{sc}^{n}$, we can get $\pi_{sc}^{r}-\pi_{sc}^{n}=\frac{4bk^{2}\eta_{r}{\left(s-bc-p_{c}eb\right)}^{2}[(1+2\eta_{r})(4bk-{\left(\lambda +p_{c}eb\right)}^{2})(2bk-{\left(\lambda +p_{c}eb\right)}^{2})-4b^{2}k^{2}\eta_{r}]}{{\left[4bk-{\left(\lambda +p_{c}eb\right)}^{2}\right]}^{2}{\left[(1+\eta_{r})(4bk-{\left(\lambda +p_{c}eb\right)}^{2})+4bk\eta_{r}\right]}^{2}}$. Let $[4bk-{\left(\lambda +p_{c}eb\right)}^{2}][2bk-{\left(\lambda +p_{c}eb\right)}^{2}]/(4b^{2}k^{2})=G$, if $G\geq \eta_{r}/(2\eta_{r}+1)$, it is easy to know that $(1+2\eta_{r})G-\eta_{r}\geq 0$. Since $2bk-{\left(\lambda +p_{c}eb\right)}^{2}>0$ and $s-bc-p_{c}eb>0$, we have $\pi_{sc}^{r}\geq \pi_{sc}^{n}$. If $G<\eta_{r}/(2\eta_{r}+1)$, and we have $\pi_{sc}^{r}<\pi_{sc}^{n}$. (iii) By comparing $\pi_{sc}^{*}$ and $\pi_{sc}^{r}$, we can get $\pi_{sc}^{*}-\pi_{sc}^{r}=\frac{2bk^{2}{\left(s-bc-p_{c}eb\right)}^{2}[2\eta_{r}{\left(\lambda +p_{c}eb\right)}^{2}(1+2\eta_{r})+bk{\left(1+\eta_{r}\right)}^{2}]}{[2bk-{\left(\lambda +p_{c}eb\right)}^{2}]{\left[(1+\eta_{r})(4bk-{\left(\lambda +p_{c}eb\right)}^{2})+4bk\eta_{r}\right]}^{2}}$. Since $2bk-{\left(\lambda +p_{c}eb\right)}^{2}>0$ and $s-bc-p_{c}eb>0$, we have $\pi_{sc}^{r}<\pi_{sc}^{*}$. Corollary 4 is demonstrated. □

## Appendix I

**Proof of Proposition** **5.** Since $\beta^{r}$, $w^{r}$, $p^{r}$ and $q^{r}$ are derived from the retailer’s fairness concern coefficient $\eta_{r}$ respectively, we can get $\frac{\delta \beta^{r}}{\delta \eta_{r}}=\frac{-4bk(\lambda +p_{c}eb)(s-bc-p_{c}eb)}{{\left[(\eta_{r}+1)(4bk-{\left(\lambda +p_{c}eb\right)}^{2})+4bk\eta_{r}\right]}^{2}}$, $\frac{\delta w^{r}}{\delta \eta_{r}}=\frac{-4bk(s-bc-p_{c}eb)[2k-p_{c}e(\lambda +p_{c}eb)]}{{\left[(\eta_{m}+1)(4bk-{\left(\lambda +p_{c}eb\right)}^{2})+4bk\eta_{m}\right]}^{2}}$,$\frac{\delta p^{r}}{\delta \eta_{r}}=\frac{-2k(s-bc-p_{c}eb)[2bk-(\lambda +p_{c}eb)(\lambda -p_{c}eb)]}{{\left[(\eta_{m}+1)(4bk-{\left(\lambda +p_{c}eb\right)}^{2})+4bk\eta_{m}\right]}^{2}}$,$\frac{\delta q^{r}}{\delta \eta_{r}}=\frac{2bk(s-bc-p_{c}eb)[2bk-{\left(\lambda +p_{c}eb\right)}^{2}]}{{\left[(\eta_{r}+1)(4bk-{\left(\lambda +p_{c}eb\right)}^{2})+4bk\eta_{r}\right]}^{2}}$. Since $2bk-{\left(\lambda +p_{c}eb\right)}^{2}>0$, it is easy to know that $2k-p_{c}e(\lambda +p_{c}eb)>0$ and $2bk-(\lambda +p_{c}eb)(\lambda -p_{c}eb)>0$. Furthermore, since $s-bc-p_{c}eb>0$, we have $\delta \beta^{r}/\delta \eta_{r}<0$, $\delta w^{r}/\delta \eta_{r}<0$, $\delta p^{r}/\delta \eta_{r}<0$ and $\delta q^{r}/\delta \eta_{r}>0$. Proposition 5 is demonstrated. □

## Appendix J

**Proof of Proposition** **6.** $\pi_{r}^{r}$ and $\pi_{m}^{r}$ can be derived from the retailer’s fairness concern coefficient $\eta_{m}$, then we can get $\frac{\delta \pi_{r}^{r}}{\delta \eta_{r}}=\frac{4bk^{2}(3\eta_{r}+1){\left(s-bc-p_{c}eb\right)}^{2}[2bk-{\left(\lambda +p_{c}eb\right)}^{2}]}{{\left[(\eta_{r}+1)(4bk-{\left(\lambda +p_{c}eb\right)}^{2})+4bk\eta_{r}\right]}^{3}}$, $\frac{\delta \pi_{m}^{r}}{\delta \eta_{r}}=\frac{-16b^{2}k^{3}\eta_{r}{\left(s-bc-p_{c}eb\right)}^{2}}{{\left[(\eta_{r}+1)(4bk-{\left(\lambda +p_{c}eb\right)}^{2})+4bk\eta_{r}\right]}^{3}}$. Since $2bk-{\left(\lambda +p_{c}eb\right)}^{2}>0$ and $s-bc-p_{c}eb>0$, we have $\delta \pi_{r}^{r}/\delta \eta_{r}>0$ and $\delta \pi_{m}^{r}/\delta \eta_{r}<0$. Proposition 6 is demonstrated. □
